# Preoperative serum thyroglobulin concentration as a predictive factor of malignancy in small follicular and Hürthle cell neoplasms of the thyroid gland

**DOI:** 10.1186/1477-7819-12-282

**Published:** 2014-09-12

**Authors:** Rok Petric, Hana Besic, Nikola Besic

**Affiliations:** Department of Surgical Oncology, Institute of Oncology, Zaloska 2, SI-1000 Ljubljana, Slovenia

**Keywords:** follicular carcinoma, Hürthle cell carcinoma, predictive factors, thyroglobulin, thyroid

## Abstract

**Background:**

Cytologic examination of a fine-needle aspiration biopsy specimen cannot distinguish between benign and malignant follicular or Hürthle cell neoplasms. Serum thyroglobulin (Tg) concentrations are higher in follicular and Hürthle cell carcinomas than in benign follicular or Hürthle cell tumors, but preoperative measurement of Tg is not recommended for initial evaluation of thyroid nodules. The aim of this study was to find out whether preoperative serum Tg concentration is a predictive factor of malignant disease in patients with a follicular or Hürthle cell neoplasm with a diameter of 2 cm or less.

**Methods:**

From 1988 to 2013, a total of 244 patients (214 female, 30 male, age range 9 to 82 years, median age 52 years) had a surgical procedure at our institute because of follicular or Hürthle cell neoplasms with a tumor diameter of 2 cm or less. In these patients a preoperative concentration of Tg was determined and Tg-autoantibodies were negative. The risk factors for malignancy were identified by a chi-square test and multivariate logistic regression.

**Results:**

The histopathologic diagnoses were carcinoma, adenoma, and benign goiter in 62 (25.5%), 115 (47%), and 67 (27.5%) patients, respectively. The median preoperative Tg concentration in benign tumors, papillary carcinomas, follicular carcinomas, and Hürthle cell carcinomas was 41, 87, 72, and 106 ng/ml (*P* = 0.05), respectively. The predictive factors for carcinoma shown by the chi-square test were: sex, thyroid volume, and preoperative Tg concentration. The independent predictors of malignancy as shown by multivariate logistic regression were: male sex (odds ratio, 2.57; *P* = 0.02), and a Tg concentration of more than 80 ng/ml (odds ratio, 2.35; *P* = 0.005).

**Conclusion:**

The independent predictors of malignancy in follicular or Hürthle cell neoplasms are sex and preoperative Tg concentration.

## Background

Thyroid nodules are a common clinical problem [1], and harbor thyroid cancer in 5% to 15% of cases [2]. A thyroid nodule is most accurately evaluated by an ultrasound-guided fine-needle aspiration biopsy (FNAB) [1]. However, in the preoperative diagnosis of follicular and Hürthle cell tumors, FNAB has limited usefulness [3–5]. In follicular and Hürthle cell tumors, malignancy is confirmed by demonstration of transcapsular or vascular invasion, which cannot be seen on cytologic slides [3, 6–9]. In follicular or Hürthle cell neoplasms, cytology cannot distinguish a malignant tumor from a benign one [8, 9]. Confirmation of malignancy is possible only with histological examination of the tumor. Therefore, patients with an FNAB-proven follicular or Hürthle cell neoplasm should be treated surgically. Unfortunately, intraoperative frozen-section analysis is not reliable and does not affect intraoperative decision-making [10, 11].

In the case of a follicular or Hürthle cell neoplasm, the American Thyroid Association guidelines recommend a lobectomy or total thyroidectomy [1]. Total thyroidectomy is preferable in patients who have bilateral nodular disease, who have large tumors (>4 cm), when marked atypia is seen on the biopsy, or who prefer to undergo bilateral thyroidectomy to avoid the possibility of requiring future surgery on the contralateral lobe [1]. A completion of thyroidectomy may be necessary if malignancy is confirmed by histologic examination after a lobectomy [1]. Reliable predictive factors for malignancy are needed, to decrease the number of thyroid reoperations [12–14].

The levels of thyroglobulin (Tg) can be elevated in many thyroid diseases [1, 15]. So, according to the European and American Thyroid Associations, preoperative Tg measurement is an insensitive and non-specific diagnostic test for thyroid cancer [1, 15]. In subjects with mild-to-moderate iodine deficiency, the serum Tg concentration correlates with the size of the goiter, the presence of multinodularity and intake of iodine [16]. Furthermore, the thyroid volume, as well as the Tg concentration, is inversely related to iodine intake [16]. However, our previous studies in patients with large follicular or Hürthle cell neoplasms (median size 3 cm) showed that measurement of the preoperative serum Tg concentration may represent a useful preoperative diagnostic test. Namely, the concentration of serum Tg was higher in follicular and Hürthle cell cancers than in benign neoplasms [12–14]. However, the majority of thyroid neoplasms are smaller than 2 cm [17]. The aim of our retrospective study was to find out if serum Tg is a predictive factor for malignancy in follicular or Hürthle cell neoplasms with a diameter of 2 cm or less.

## Methods

### Patients

A chart review of 244 patients (214 female, 30 male, age range 9 to 82 years, median age 52 years) was performed. These patients had a surgical procedure at our institute from 1988 to 2013 because of follicular or Hürthle cell neoplasms with a diameter of 2 cm or less. In all patients, the preoperative concentration of Tg was determined and a test for Tg-autoantibodies was negative. The clinical data of these patients are presented in Table 1. All patients (*N* = 38) with Tg-autoantibodies were excluded from our analysis.

**Table 1:** **Clinical data of 244 patients with follicular and Hürthle cell neoplasms with diameter 2 cm or less**

Characteristic	Subgroup	Benign *N* = 182 (%)	Carcinoma *N* = 62 (%)	Chi-square test *P*value
**Age (years)**	45 or younger	57 (24%)	23 (9%)	0.44
	Older than 45	125 (51%)	39 (16%)	
	65 or younger	163 (67%)	54 (22%)	0.64
	Older than 65	19 (8%)	8 (3%)	
**Sex**	Male	17 (7%)	13 (5%)	0.024
	Female	165 (68%)	49 (20%)	
**Dominant tumor**	In multinodular goiter	61 (25%)	23 (9%)	0.64
	Solitary	121 (50%)	39 (16%)	
**Tumor diameter (cm)**	1 or less	42 (17%)	13 (5%)	0.86
	More than 1	140 (58%)	49 (20%)	
**Thyroid volume (ml)**	29 or less	149 (61%)	47 (19%)	0.36
	30 or more	33 (14%)	15 (6%)	
	49 or less	169 (70%)	52 (21%)	0.045
	50 or more	13 (5%)	10 (4%)	
**Serum Tg concentration (ng/ml)**	79 or less	120 (49%)	28 (11%)	0.004
	80 or more	62 (26%)	34 (14%)	
	299 or less	168 (69%)	51 (21%)	0.031
	300 or more	14 (6%)	11 (4%)	

All patients had a follicular or Hürthle cell neoplasm diagnosed by FNAB and cytology. FNABs were ultrasound-guided in the majority of cases. FNABs were performed using a 21-23 gage needle attached to a 10 ml syringe. All cytologic slides were examined by cytopathologists and histologic slides by pathologists, all of them experienced in thyroid pathomorphology. Routine cytologic and final pathology reports were used in this study.

Clinically, the dominant nodule was either solitary or in a multinodular goiter in 160 and 84 cases, respectively (Table 1). The sizes of the thyroid gland and the dominant nodule were determined on the basis of measurements performed during the pathologic examination of the biopsy material. The thyroid volume was calculated by summing the volumes of both lobules. The volume of each lobe was calculated using the formula:

as already reported [14].

Our study was reviewed and approved by the Institutional Review Board of the Institute of Oncology, Ljubljana. It was performed in accordance with the ethical standards laid down in an appropriate version of the 1964 Declaration of Helsinki. Our study was conducted with the understanding and consent of the subjects. All our patients were asked during first admission to our institute or a follow-up visit to give consent for the study of their charts and biopsy material for scientific purposes. Since the Institutional Review Board of the Institute of Oncology Ljubljana approved this specific study, our patients were not asked to give written consent on this specific study.

### Laboratory

From 1988 to 2001 and from 2007 to 2008, serum Tg measurements were carried out using the commercially available Dyno-test Tg/Tg-S RIA kit (Henning/Brahms, Germany), which includes the determination of Recovery-Tg. Normal values of Recovery-Tg were considered to rule out the possibility of disturbing influence exerted by Tg-autoantibodies or other non-specific serum proteins. From 2001 to 2007, serum Tg and Tg-autoantibody concentrations were determined by the commercially available LIAISON Tg and LIAISON Anti-Tg kits, respectively, with the LIAISON analyzer (all from Byk-Sangtec Diagnostica/DiaSorin, Germany/Italy). From 2008 onwards, serum Tg and Tg-autoantibody concentrations were determined by the commercially available Tg/Tg-II Cobas and Anti-Tg kits, respectively, with a Modular analyzer (all from Roche, Germany).

Owing to the presence of Tg-autoantibodies, 38 patients were excluded from the study.

### Statistical analysis

The predictive factors for malignancy were identified by a chi-square test. Only the status of the dominant nodule was used for the analysis of factors correlating with the presence of malignancy. A receiver operating characteristic (ROC) analysis was performed to define the cut-off for Tg levels. A cut-off for Tg values was chosen at 80 ng/ml on the basis of a ROC curve. The sensitivity, specificity, and positive and negative predictive values of elevated Tg concentrations for the presence of carcinoma were calculated. The following factors were included in the univariate and multivariate statistical analyses: the sex of the patients, volume of the thyroid gland, and Tg concentration. The variables that showed statistical correlation (*P* < 0.05) by logistic regression analysis were considered as independent predictive factors for malignancy. SPSS 16.0 for Windows was used for statistical analysis.

## Results

The median age of the patients, tumor size, volume of the thyroid gland, median and mean concentration of preoperative Tg were 52 years (range, 9 to 82), 1.5 cm (range, 0.4 to 2), 20 ml (range, 5 to 162), 50 ng/ml (range, 1 to 3,424), and 125 (range 1 to 3,424) ng/ml, respectively. The histopathologic diagnoses were carcinoma, adenoma, and benign goiter in 62 (25.5%), 115 (47%), and 67 (27.5%) patients, respectively.

**Figure 1:**
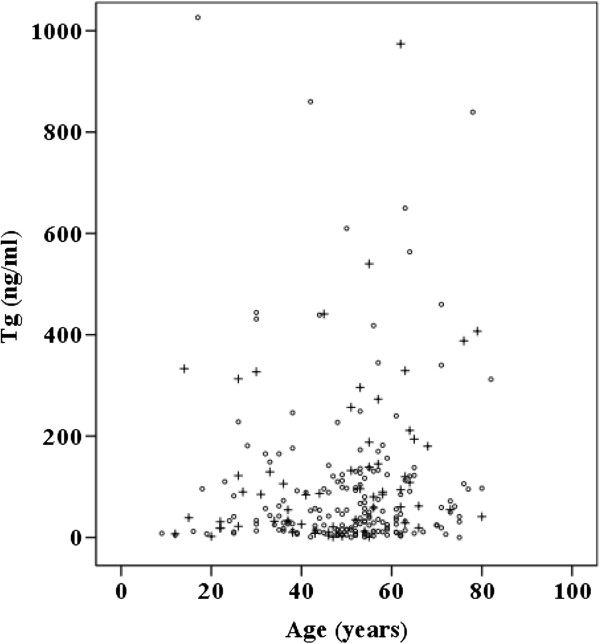
**Serum Tg concentration and age of patients with follicular and Hürthle cell neoplasms.** +, with carcinoma; O, without carcinoma.

**Figure 2:**
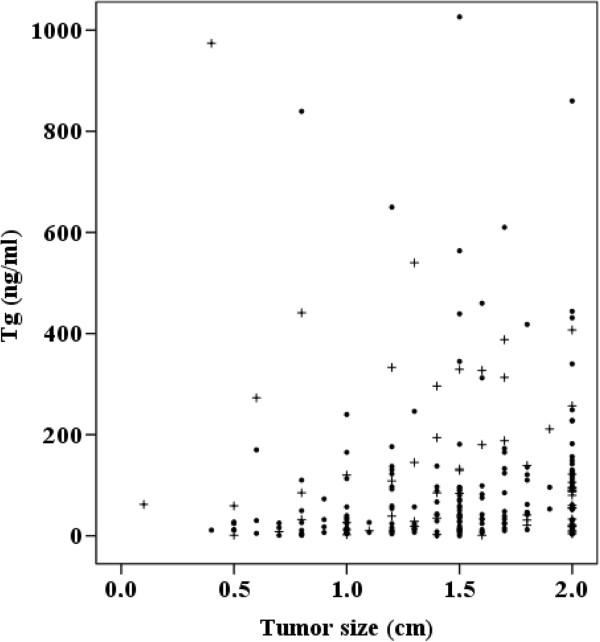
**Serum Tg concentration and tumor size in follicular and Hürthle cell neoplasms.** +, with carcinoma; O, without carcinoma.

The median age of patients with carcinoma was 52.5 years, while the median age of those with a benign disease was 52 years. The median size of the tumor was 1.55 cm in patients with carcinoma and 1.5 cm in those with a benign disease. Tumor diameter did not correlate with malignancy rate (*P* = 0.225). These data are presented in Table 2 and Figures 1 and 2.

**Table 2:** **Tumor size, presence of carcinoma, and Tg concentration**

Tumor size (cm)	Number of patients	Benign tumor median (range) Tg concentration ng/ml	Carcinoma median (range) Tg concentration ng/ml	*P*value
**0.01 to 0.5**	9	12 (11 to 27)	60 (1 to 974)	0.225
**0.51 to 1**	46	18 (1 to 839)	32 (2 to 441)	
**1.01 to 1.5**	83	41 (2 to 1,026)	119 (3 to 3,424)	
**1.51 to 2**	106	62 (3 to 860)	90 (1 to 1,757)

The median preoperative Tg concentrations in patients with and without carcinoma were 86 and 41 ng/ml, respectively. The mean preoperative Tg concentrations in patients with and without carcinoma were 215 and 95 ng/ml, respectively. The median preoperative Tg concentrations in benign tumors, papillary carcinomas, follicular carcinomas, and Hürthle cell carcinomas were 41, 87, 72, and 106 ng/ml (*P* < 0.05), respectively. The mean preoperative Tg concentrations in benign tumors, papillary carcinomas, follicular carcinomas, and Hürthle cell carcinomas were 95, 147, 433, and 269 ng/ml (*P* < 0.001), respectively. Tg concentrations in different types of carcinoma in the dominant nodule are listed in Table 3. As shown by logistic regression analysis, concentration of Tg (cut-off point of 80 ng/ml) was an independent predictive factor. The ROC analysis showed that the area under the curve was 0.613 (*P* = 0.008). The sensitivity and specificity of the test, positive predictive value, and negative predictive value (cut-off = Tg concentration of 80 ng/ml) for the presence of carcinomas in small follicular and Hürthle cell neoplasms of the thyroid gland were 54.8%, 31.1%, 35.4%, and 81%, respectively.

**Table 3:** **Histology of follicular and Hürthle cell neoplasms and Tg concentration**

Histology	Number of patients	Tg concentration ng/ml (range)	Tg concentration ng/ml (median)
**Carcinoma**	**62**	**1 to 3,424**	**86**
Follicular	10	2 to 3,424	72
Hürthle cell	11	1 to 1,757	106
Papillary, classic type	9	1 to 974	85
Papillary, follicular type	31	1 to 540	90
Papillary, Hürthle cell	1	4	4
**Benign tumor**	**182**	**1 to 1,203**	**41**
Adenoma	115	1 to 1,026	32
Benign goiter	67	1 to 839	54

Benign tumors versus papillary carcinomas and versus follicular or Hürthle cell carcinomas (*P* < 0.001).

Predictive factors for malignancy in follicular or Hürthle cell neoplasms as shown by the chi-square test were: sex, volume of the thyroid gland, and Tg concentration (Table 1). The independent predictors of malignancy as shown by multivariate logistic regression were: sex and preoperative Tg concentration (Table 4). Male patients had a higher risk of carcinoma than female patients (odds ratio, 2.57). In male patients, the malignancy rate was 43%, while in female patients, it was 23%. Patients with a preoperative Tg concentration of 80 ng/ml or higher had a higher risk of malignancy than those with a lower concentration (odds ratio, 2.35). In patients with a Tg concentration of 80 ng/ml or more, the malignancy rate was 35%, while in those with a smaller Tg concentration, it was 19%.

**Table 4:** **Independent risk factors for malignancy in follicular or Hürthle cell neoplasms**

Factor	Subgroup	Odds ratio	95% confidence interval
**Sex**	Female	1	1.15 to 5.75
	Male	2.57	
**Serum Tg concentration (ng/ml)**	79 or less	1	1.30 to 4.25
	80 or more	2.35	

The likelihood of malignancy as calculated by logistic regression (model summary: chi-square: 13.747; 2 degrees of freedom; *P* = 0.002; -2 log likelihood of 263.32).

Incidental carcinoma in a non-dominant tumor was detected in 13 patients. It was detected in patients with a benign disease and carcinoma of the dominant nodule in 9 (5%) and 4 (6%) of cases, respectively. In patients with carcinoma in the dominant nodule, final pathology additionally revealed a benign goiter, benign adenoma, and thyroiditis in 21, 10, and 27 cases, respectively.

## Discussion

The following predictive factors of malignancy have been reported in the literature [5, 13, 14, 17–41]: the patient’s age [18, 19, 24, 25, 30–33] and sex [5, 26], the tumor size [5, 18, 20–22, 24–26, 31–36, 42], a solitary nodule [24, 26, 27], tumor fixation [25], a lesion that feels hard on palpation [27], Tg concentration [13, 14, 18], ultrasound characteristics [17, 27–29, 41], cytologic characteristic [21], and gene expression diagnostic markers [37–40]. Ultrasound characteristics predictive of malignancy are: the presence of internal blood flow (as detected by Doppler ultrasonography), the presence of hypervascularity, a solid echo structure, a hypoechoic lesion, or microcalcifications [17, 27–29, 41]. New cytologic characteristics were also reported as factors predictive of malignancy: transgressing vessel [21] and nuclear grooves [21]. New gene expression diagnostic markers of malignancy are: miR-100, miR-125b, miR-138, miR-768-3p, miRNA-885-5p, and a combination of genes PVALB and C1orf24 [37–40]. However, a vast majority of reports on predictive factors in follicular or Hürthle cell neoplasms included only a small number of patients; thus, the authors of these studies could perform only a univariate statistical analysis. To our knowledge, so far only eight publications [13, 14, 17, 18, 21, 25, 26, 41] have reported the results of multivariate logistic regression analysis of predictive factors in patients with follicular or Hürthle cell neoplasms.

Carcangiu *et al.* [30], Lopez-Penabad *et al.* [31], Taneri *et al.* [32], and Zhang *et al.* [33] stated that patients with carcinoma were older than those with a benign disease. However, the median age of our patients with carcinoma was not statistically different from the median age of those with a benign disease (52.5 years versus 52 years). Our findings are in agreement with the majority of reports from the literature.

Mihai *et al.* [5] and Tuttle *et al*. [26] found that the risk of carcinoma was higher in men than in women. This is in agreement with the findings of our study. In our study, men had a higher risk of carcinoma than women (an odds ratio of 2.57). The malignancy rate in men in our study was 43%, while in women it was 23%.

Many authors reported that the size of follicular neoplasms increases the risk of malignancy [5, 18, 20–22, 24–26, 28, 31–36, 42]. Tuttle *et al.* [26] reported that the risk of carcinoma in a tumor larger than 4 cm was 40%, while in smaller tumors it was only 13%. In our patients with a tumor diameter of 2 cm or less, the risk of carcinoma was 25.5%. In Hürthle cell neoplasms, Taneri *et al.* [32] found that there was no malignancy among the tumors less than 1 cm in diameter. But our findings are quite the opposite. In our patients, the malignancy rate did not differ in patients with a tumor diameter of 1 cm or less versus bigger tumors (Table 1).

Hocevar and Auersperg [12] found that preoperative Tg concentration was higher in patients with follicular and Hürthle cell carcinoma than in patients with benign tumors. In Hürthle cell neoplasms, Sugino *et al.* [42] reported that in patients with carcinoma and benign disease the median Tg concentration was 1,782 ng/ml and 573 ng/ml, respectively. Strazisar *et al.* [14] and Petric *et al.* [13] reported that there was a statistical difference in preoperative Tg concentrations of benign tumors, papillary carcinomas, follicular carcinomas, and Hürthle cell carcinomas. Moreover, the results of the present study show that the mean preoperative Tg concentration was significantly different (*P* < 0.05) in benign tumors, papillary carcinomas, follicular carcinomas, and Hürthle cell carcinomas. The median preoperative Tg concentrations in benign tumors, papillary carcinomas, follicular carcinomas, and Hürthle cell carcinomas were 41, 87, 72, and 106 ng/ml, respectively. However, in contrast to our findings, Suh *et al.* [23] found, in only 39 patients with follicular or Hürthle cell neoplasms, that Tg was not an independent risk factor [23]. The median preoperative levels of the Tg serum were, in patients with malignant and in patients with benign tumors, 135 ng/ml and 116 ng/ml, respectively [23]. Their patients had bigger tumors than did the patients from our present study. Their patients’ Tg concentrations were larger than ours, probably because the median tumor size of their patients was bigger than in our study. The median diameter of their malignant and benign tumors was 2.8 cm and 2.4 cm, respectively.

The aim of this study was to find out whether Tg is a predictive factor of malignancy in 244 small (2 cm or less) follicular or Hürthle cell neoplasms. In our study, the concentration of Tg was an independent predictive factor of malignancy. Patients with a preoperative Tg concentration of at least 80 ng/ml had a higher risk of malignancy than those with a lower concentration (an odds ratio of 2.35). In patients with a Tg concentration of 80 ng/ml or more, the malignancy rate was 35%, while in those with a smaller Tg concentration, it was only 19%. We believe that determination of Tg concentration may be one of the factors that are useful in follicular or Hürthle cell neoplasms before determining the extent of the thyroid surgical procedure.

## Conclusions

The malignancy rate in this study of 244 patients with follicular or Hürthle cell neoplasms with a tumor diameter of 2 cm or less was 25.5%. The independent predictors of malignancy in follicular or Hürthle cell neoplasms are: sex and preoperative Tg concentration.

## Abbreviations

FNAB: fine-needle aspiration biopsy
ROC: receiver operating characteristic
Tg: thyroglobulin.
